# Non-odontogenic tumors of the facial bones in children and adolescents: role of multiparametric imaging

**DOI:** 10.1007/s00234-017-1798-y

**Published:** 2017-03-13

**Authors:** Minerva Becker, Salvatore Stefanelli, Anne-Laure Rougemont, Pierre Alexandre Poletti, Laura Merlini

**Affiliations:** 1 Division of Radiology, Department of Imaging and Medical Informatics, Geneva University Hospital, University of Geneva, Rue Gabrielle Perret Gentil 4, 1211 Geneva, Switzerland; 2 Division of Clinical Pathology, Department of Genetic and Laboratory Medicine, Geneva University Hospital, University of Geneva, Geneva, Switzerland

**Keywords:** Head and neck, Tumors, Facial bones, Multimodality and multiparametric imaging

## Abstract

Tumors of the pediatric facial skeleton represent a major challenge in clinical practice because they can lead to functional impairment, facial deformation, and long-term disfigurement. Their treatment often requires a multidisciplinary approach, and radiologists play a pivotal role in the diagnosis and management of these lesions. Although rare, pediatric tumors arising in the facial bones comprise a wide spectrum of benign and malignant lesions of osteogenic, fibrogenic, hematopoietic, neurogenic, or epithelial origin. The more common lesions include Langerhans cell histiocytosis and osteoma, while rare lesions include inflammatory myofibroblastic and desmoid tumors; juvenile ossifying fibroma; primary intraosseous lymphoma; Ewing sarcoma; and metastases to the facial bones from neuroblastoma, Ewing sarcoma, or retinoblastoma. This article provides a comprehensive approach for the evaluation of children with non-odontogenic tumors of the facial skeleton. Typical findings are discussed with emphasis on the added value of multimodality multiparametric imaging with computed tomography (CT), magnetic resonance imaging (MRI) with diffusion-weighted imaging (DWI), positron emission tomography CT (PET CT), and PET MRI. Key imaging findings and characteristic histologic features of benign and malignant lesions are reviewed and the respective role of each modality for pretherapeutic assessment and post-treatment follow-up. Pitfalls of image interpretation are addressed and how to avoid them.

## Introduction and background

Tumors arising in the pediatric facial skeleton represent a formidable challenge in clinical practice because of the complex anatomy of the head and neck (HN) and because of facial development during this time span. Although rare, these conditions include a broad spectrum of lesions with a varying degree of malignant potential [1–4]. Their true incidence is difficult to establish due to the paucity of reports in the literature and because most published articles are either isolated case reports or series dealing with a specific histological diagnosis. Depending on lesion origin, tumors can be classified as primarily originating in the facial bones or as metastases to the facial skeleton. Tumors arising within the jaws can be further subdivided into odontogenic and non-odontogenic. Most pediatric tumors of the facial bones are of non-odontogenic origin [2, 3]. They tend to occur in the mandible in the 11–15-year-old age group, and the majority (70–90%) are benign [2, 3].

Symptoms are often non-specific and include swelling, facial asymmetry, induration, pain, paresthesia, bleeding, or increased tooth mobility [1]. However, due to the slow growth rate, most lesions do not cause pain. In a recent article reviewing the clinical presentation, management, and outcome of children with odontogenic and non-odontogenic jaw tumors seen over a period of 20 years, the authors found that as many as 25% of patients were asymptomatic, the tumors being diagnosed only by imaging [1]. Irrespective of origin and dignity, prompt workup with cross-sectional imaging and—whenever necessary—biopsy are crucial for a successful multidisciplinary management [1–4]. Multidisciplinary treatment requires a close collaboration between pediatricians, radiologists, medical oncologists, maxillofacial and HN surgeons, radiotherapists, and pathologists. In addition, long-term follow-up is necessary in many pediatric cases because of subsequent facial deformity or functional impairment at the adult age. Facial deformity and functional impairment may be the result of asymmetric facial growth related to the lesion itself or to its treatment.

Radiologists evaluating children with facial lesions must be aware of the typical imaging findings of benign and malignant tumors and their mimics, characteristic patterns of tumor spread, and potential pitfalls of image interpretation; furthermore, they must be confident when recommending biopsy or a “wait-and-see” policy as biopsy may result in unnecessary morbidity whereas a wait-and-see policy may prove to be hazardous. In order to achieve these goals, multimodality imaging is often required and integration of multiple and sometimes discordant findings into a comprehensive radiological report is essential.

To the best of our knowledge, a comprehensive review of multiparametric and multimodality imaging features of these rare tumors has not yet been published. The purposes of this article are to review typical imaging findings and discuss the added value of multimodality imaging with computed tomography (CT), magnetic resonance imaging (MRI) including diffusion-weighted imaging (DWI), positron emission tomography CT (PET CT), and PET MRI. We highlight key imaging features of benign and malignant tumors based on radiologic-pathologic correlation, and we discuss potential pitfalls of image interpretation caused by overlapping imaging features.

## Imaging techniques

High-resolution, thin-slice MRI and CT are the standard of care for the assessment of facial lesions, whereas ultrasonography (US) is the preferred method for evaluating superficial soft tissue swellings caused by extracranial HN masses. The choice of the most appropriate imaging technique depends on the individual clinical status: a soft, mobile, well-delineated, and compressible swelling will often be imaged with US first, whereas an indurated, poorly defined soft tissue lesion adherent to bone will be imaged with MRI and/or CT. MRI is preferred over CT because of its higher soft tissue contrast and higher specificity. The MRI protocol should routinely include T1- and T2-weighted sequences with or without fat saturation and a DWI sequence with at least two *b* values (*b* = 0 and *b* = 1000) for mono-exponential fitting. The apparent diffusion coefficient (ADC) is a metric used for quantification. In the extracranial HN, restricted diffusion (high signal on *b* 1000 and low ADC values) is typically seen in malignant hypercellular tumors, in abscesses, in thrombi, and in Warthin tumors, whereas the absence of restriction is the hallmark of benign tumors. The reported mean ADC values for malignant pediatric tumors are below 0.8 × 10^−3^ mm^2^/s, whereas benign tumors (with the exception of fibroma) have ADC values in the range of 1.25–1.6 × 10^−3^ mm^2^/s; the ADC values of cystic lesions are in the range of 1.9–2.2 × 10^−3^ mm^2^/s [5]. Contrast-enhanced MRI (CEMRI) images should be acquired whenever a tumor or a vascular lesion is suspected, to improve lesion characterization and to detect perineural spread. Nevertheless, as the information obtained by MRI and CT is often complementary, our institutional practice includes an MRI examination (if possible without sedation) followed by a low-dose CT protocol to assess bony structures for improved lesion characterization or for presurgical planning. As suggested in the literature [6], the radiation dose delivered by CT should be minimized in accordance to the As Low As Reasonably Achievable (ALARA) principle, in particular as children have a higher risk for expressing radiation effects during their life span than adults [6]. Therefore, whenever CT is needed, the size of the scanned body region should be limited to the minimum necessary and a low-dose protocol should be applied. Due to its low radiation dose and high resolution for bony structure abnormalities, cone beam CT (CBCT) can be used as an alternative to CT. However, CBCT scans with adult settings may result in excessive radiation to children scanned with such default adult settings [7]. Therefore, collimation should be used whenever possible to reduce the radiation dose. As with CT, the use of CBCT scans should be justified on a case-by-case basis [7].

F^18^-Fluoro-deoxy-d-glucose (FDG) PET CT is a rapid and reliable technique for the initial assessment and follow-up of pediatric and adult tumors originating in the HN [8–13]. As FDG is taken up by all cells with an increased glucose metabolism, uptake in the normal lymphoid tissue of Waldeyer’s ring, salivary glands, and brown fat, as well as inflammatory and infectious conditions or granulomatous diseases, may occasionally lead to false-positive assessments [13]. On the contrary, small lesion size and vicinity to anatomic areas with high-glucose metabolism, such as the brain, can lead to false-negative interpretations unless correlation with MRI or CT is obtained [13]. As an alternative to PET CT, the recently introduced hybrid PET MRI technology has emerged as a promising tool in adult and pediatric oncology [14–17]. It has the advantage of offering decreased radiation exposure in addition to combined anatomic, functional, and metabolic information. PET MRI is technically feasible in children as young as 6 years of age without general anesthesia, showing comparable accuracy to PET CT [17]. However, child-specific patient preparation procedures are mandatory in order to obtain good-quality examinations. Staging and follow-up of pediatric lymphoma have been recognized as a key application of whole-body PET MRI [17, 18].

Advanced post-processing techniques are gaining increasing popularity for presurgical assessment, for treatment planning, and for post-treatment follow-up. Fusion of PET and CT data is routinely performed to combine anatomic and metabolic information. Fusion of PET or DWI images and anatomic MRI sequences can be easily obtained with hybrid PET MRI systems, whereas, if PET data are acquired independently from MRI data, multimodality fusion can be obtained by commercially available software algorithms. These algorithms also enable fusion of MRI and CT data to enhance simultaneous soft tissue and osseous visualization. Tumor volumetry can be obtained with semiautomated or automated segmentation methods, and multilayer 3D reconstructions depicting the tumor, major arteries, and veins, as well as skull base foramina and cranial nerves, are further examples of advanced post-processing techniques. In our institution, we routinely perform these techniques whenever complex craniofacial tumor resection is planned.

## Primary intraosseous tumors

Primary intraosseous tumors of non-odontogenic origin arising in children and adolescents include osteoma and osteoblastoma, Langerhans cell histiocytosis, desmoplastic fibroma, inflammatory myofibroblastic tumor, juvenile ossifying fibroma, Ewing sarcoma and osteosarcoma, and primary intraosseous lymphoma.

### Osteoma

**Fig. 1:**
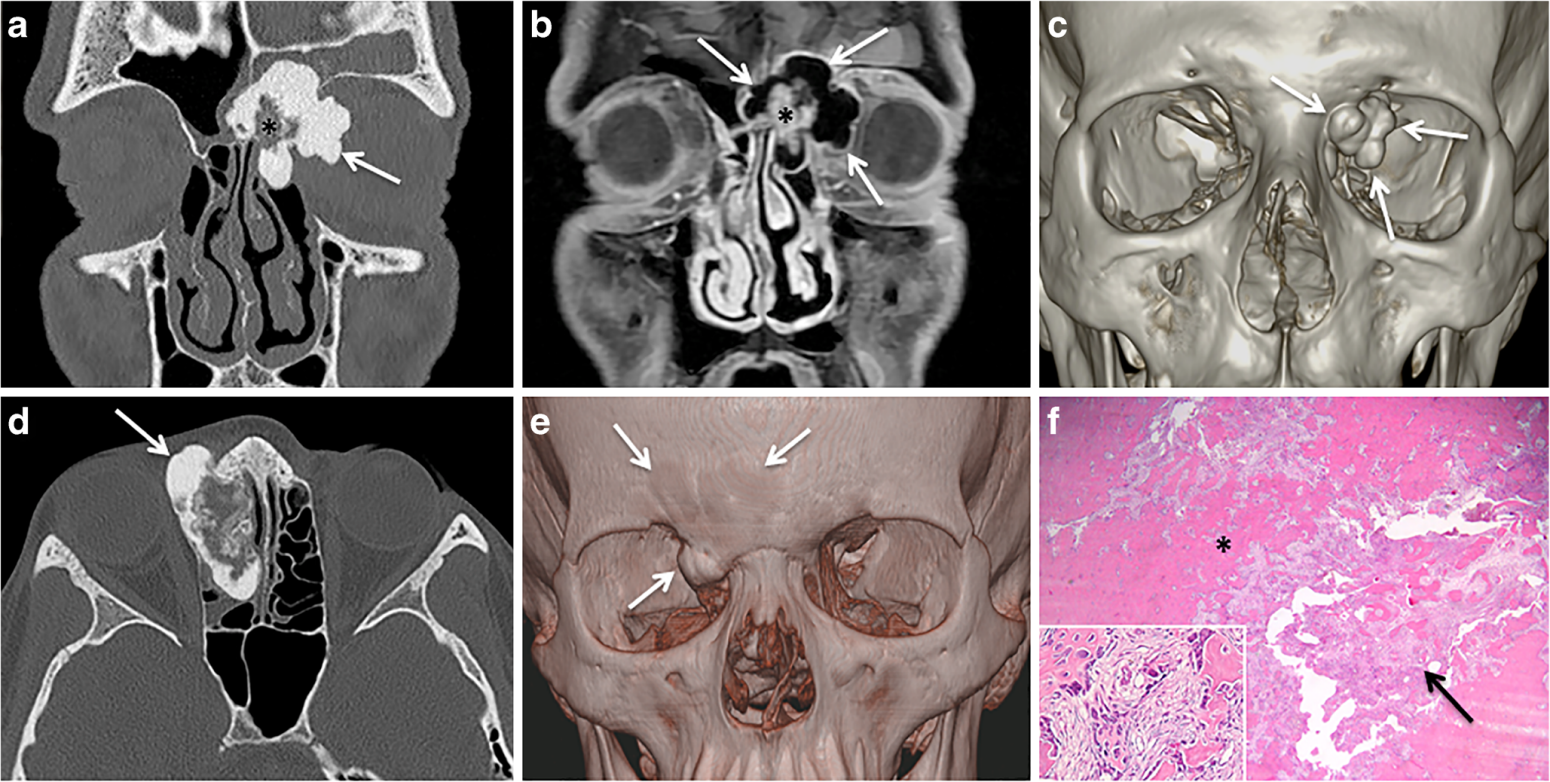
Two histologically proven cases of osteoma. Ethmoid sinus osteoma in a 15-year-old boy with left orbital pain and recurrent sinusitis since 6 months. **a** Coronal CT scan (bone window) shows a well-circumscribed mass (*arrow*) with ossification of variable density. The peripheral dense portion corresponds to ivory osteoma (*arrow*), whereas the central portion (*asterisk*) corresponds to trabecular osteoma. **b** Corresponding coronal fat-saturated T1 obtained after iv. gadolinium. The central osteoma portion (trabecular bone with marrow) enhances after contrast material (*asterisk*). In the absence of CT, the ivory osteoma portion can be misinterpreted as air. **c** 3D CT reconstruction: left intraorbital extension of the ossified polylobulated mass (*arrows*). Ethmoid sinus osteoma with osteoblastoma-like features in a 12-year-old girl with right orbital pain and diplopia. **d** Axial CT scan (bone window) shows a well-circumscribed polylobulated mass (*arrow*) with a very dense peripheral portion and a central area of ground glass opacity. **e** 3D CT reconstruction: left intraorbital extension of the ossified polylobulated mass and involvement of the frontal bone (*arrows*). **f** Low-power photomicrograph after complete resection (original magnification, ×20; hematoxylin-eosin [H-E] stain) shows the central nidus-like area (*arrow*) surrounded by an outer rim of sclerotic cortical bone (*asterisk*). At high power, *inset* in **f**, the nidus-like portion is composed of interconnected woven bone and osteoid, lined by plump osteoblasts (original magnification, ×200; H-E stain)

**Fig. 2:**
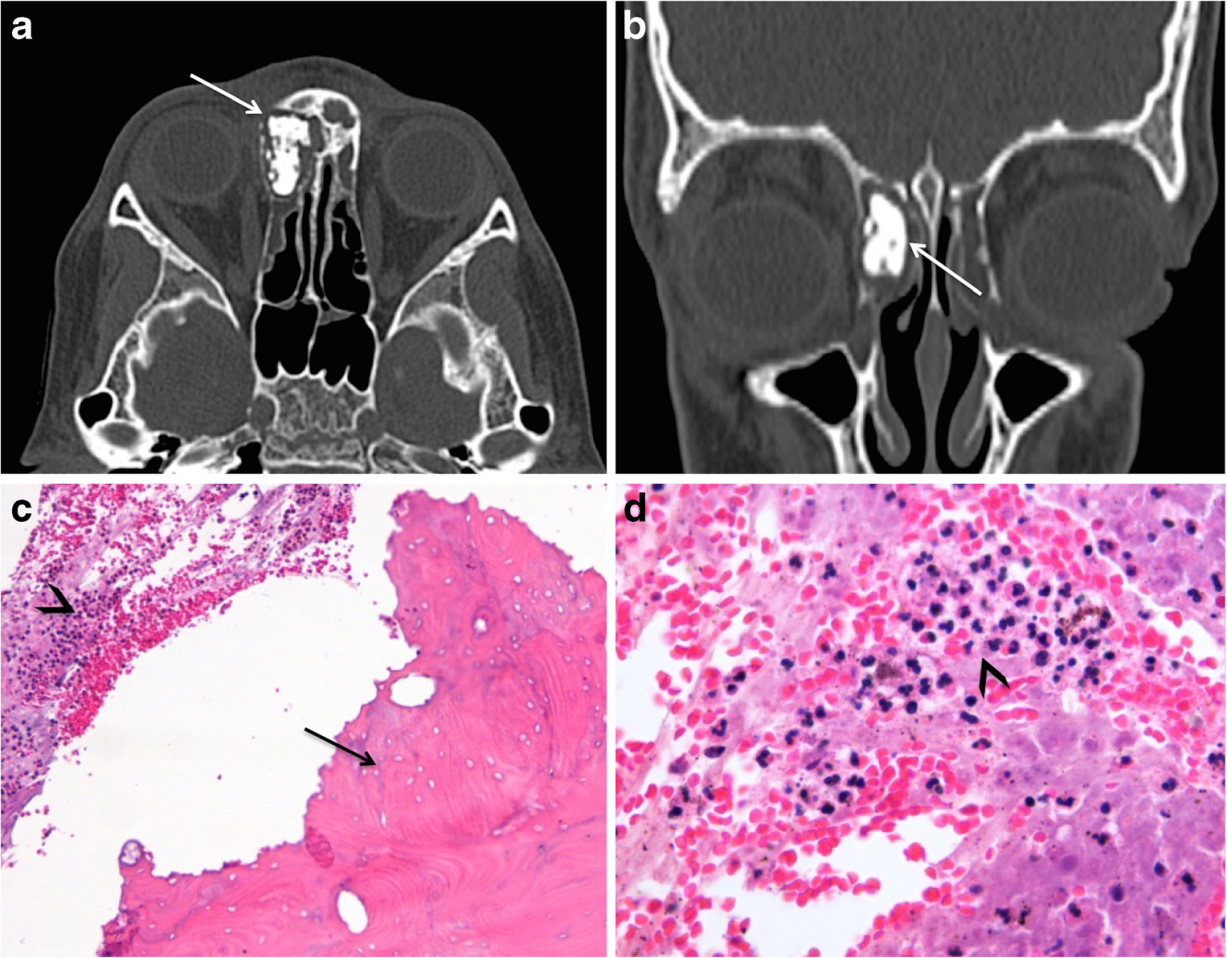
Large bone sequestrum in osteomyelitis mimicking ivory osteoma in a 13-year-old boy with recurrent sinusitis and right eyelid and frontal swelling since several months. **a** Axial and **b** coronal CT images (bone window) show a well-circumscribed lesion of bone density (*arrows*) in the right ethmoidal cells. The diagnosis of ivory osteoma was suggested. Histology (**c**, original magnification, ×200; H-E stain) and (**d**, original magnification, ×400; H-E stain), however showed osteomyelitis with a large devitalized bone fragment corresponding to a sequestrum (*arrow* in **c**), surrounded by neutrophils and necrotic debris (*arrowheads* in **c**, **d**)

Craniofacial osteomas are benign, slowly growing tumors that are mainly seen in the paranasal sinuses, jaws, and outer table of the skull vault. They represent 3% of all benign paranasal sinus tumors and are most often encountered after 40 years of age. Although osteomas are commonly found on CT examinations of the paranasal sinuses in adults, they are very uncommon in the pediatric population [19, 20]. The etiology of osteoma is unclear, and developmental, traumatic, or infective origins have been suggested [20]. Multiple osteomas are part of Gardener syndrome. Histologically, osteomas can be divided into (1) ivory or compact osteomas composed of mature lamellar bone without Haversian canals and without fibrous components and (2) trabecular or spongious osteomas composed of cancellous trabecular bone surrounded by a cortical bone margin. Some tumors show mixed or overlapping features. Most osteomas are usually smaller than 20 mm in diameter although giant osteomas (>30 mm) have been reported [21]. Clinically, most craniofacial osteomas are asymptomatic and they are usually detected by cross-sectional imaging. However, when large, they may result in facial asymmetry, pain (due to nerve compression), headache, diplopia, exophthalmos and excessive lacrimation (due to extension into the orbit), sinusitis and mucocele (due to obstruction of the sinonasal drainage pathways), cerebrospinal fluid rhinorrhea, and dental malocclusion and trismus (when the mandible or maxilla is involved). In children and adolescents, craniofacial osteomas tend to arise in the frontal or ethmoid sinuses at the junction between endochondral and membranous bones. According to the literature, nearly 40% of sino-orbital osteomas have histologic features similar to osteoblastomas (immature bone with enlarged osteoblasts and fibrovascular stroma); therefore, the term “sino-orbital osteoma with osteoblastoma like features” has been recommended [22]. Some authors have even suggested that probably many reported sino-nasal osteoblastomas are actually osteomas with osteoblastoma-like features [23]. The CT and MRI aspect of sino-nasal osteoma is straightforward and reflects the underlying histology (Fig. 1). On CT, ivory osteomas characteristically appear as very dense calcified lesions, whereas trabecular osteomas may demonstrate bony marrow (Fig. 1). Occasionally, osteomas are less dense and they display a ground glass appearance at CT. As the ivory osteoma portions tend to have very low signal intensity on all MRI sequences, in the absence of CT, these low-signal-intensity areas can be misinterpreted as air within the paranasal sinuses or nasal fossae (Fig. 1). Correlation with CT or CBCT is, therefore, mandatory. In trabecular osteomas, due to the variable presence of fatty marrow and red marrow, the signal intensity can be quite variable on T1-weighted and T2-weighted images, whereas after administration of gadolinium chelates, moderate to strong enhancement is observed. In rare cases, chronic osteomyelitis with button sequestrum (devascularized bone surrounded by a lucent rim) can mimic osteoma at cross-sectional imaging and only histologic analysis allows the correct diagnosis (Fig. 2). As opposed to osteomas, which typically present polypoid intraorbital or intrasinonasal growth, osteoid osteoma, which is exceptionally rare in the craniofacial bones, presents with a central nidus surrounded by a sclerotic peripheral zone [22]. Surgery via an endoscopic approach is the treatment of choice in large osteomas and osteoblastomas while small asymptomatic osteomas are followed clinically and with cross-sectional imaging. As endoscopic procedures tend to be more difficult in children due to the small anatomic structures, a neuronavigation-guided endoscopic procedure while being prepared to perform an open approach is preferred [20]. More recently, radiofrequency ablation has been shown to provide safe, minimally invasive, and effective long-lasting treatment of osteoid osteoma arising in the spine, periarticular/intraarticular, and lower limb regions [24]. Although this technique is associated with reduced health care resources and although it has been recommended as the treatment of choice for osteoid osteoma of the limbs in children and adolescents [25], its use in osteomas arising in the craniofacial skeleton has not been reported. CT and CBCT are recommended for follow-up, if necessary.

### Langerhans cell histiocytosis

**Fig. 3:**
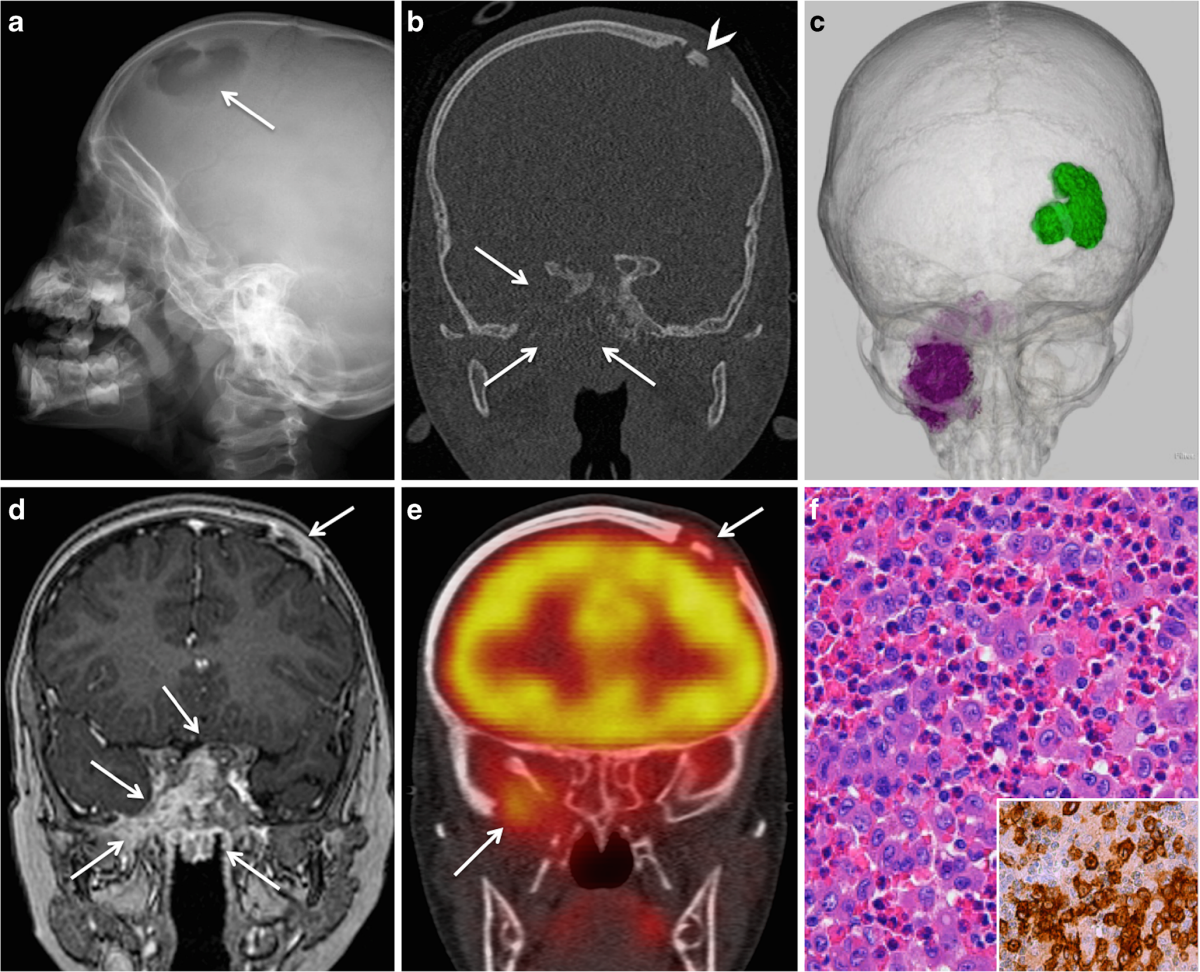
Craniofacial lesions in LCH in a 4-year-old boy with fever, failure to thrive, and diabetes insipidus. **a** Conventional X-ray of lateral skull shows a large lytic calvarial lesion (*arrow*). **b** Coronal CT scan (bone window) shows lytic calvarial lesion with button sequestrum (*arrowhead*), as well as extensive osteolysis of the sphenoid bone (*arrows*). **c** 3D CT reconstruction: calvarial lesion in *green* and base of the skull lesion in *purple*. **d** Coronal contrast-enhanced T1 shows homogeneous and strong enhancement of the destructive lesions (*arrows*). Invasion of the pterygopalatine fossa, pterygomaxillary fissure, greater wing of the sphenoid, and adjacent dura and extension into the nasopharynx (*arrows*). **e** Coronal FDG PET/CT image shows focal FDG uptake of both lesions (*arrows*). SUVmean/SUVmax were 3.09/3.90 and 3.26/4.55, respectively. No other lesions were found in the rest of the body neither on whole-body MRI nor on PET/CT. **f** High-power photomicrograph (original magnification, ×400; H-E stain) shows sheets of Langerhans cells with indented vesicular nuclei, admixed with numerous eosinophils. Immunohistochemistry (*inset* in **f**, original magnification, ×400) reveals strong reactivity of Langerhans cells to CD1A (brown reaction product). There was also strong reactivity to S100 protein (not shown)

Langerhans cell histiocytosis (LCH) is a rare disease with an incidence of 0.5–5 cases per million persons per year [26–28]. It comprises three entities: (1) eosinophilic granuloma (EG), which is the benign unifocal form of LCH; (2) Hand-Schüller-Christian triad, which is the combination of diabetes insipidus, lytic bone lesions, and exophthalmos; and (3) multifocal multisystem LCR, also called Abt-Letterer-Siwe disease, which is a fulminant disease with abdominal involvement and poor prognosis. The pathogenesis of LCH is not known, and some arguments support the reactive nature while other arguments, such as the presence of BRAF V600E and MAP2K1 gene mutations, rather suggest a neoplastic process [28]. LCH is caused by clonal proliferation of activated dendritic cells and macrophages. Cells often express S100, CD1a, and CD2017/Langerin (Fig. 3). Characteristic Birbeck granules are seen at electron microscopy.

LCH affects both children and adults, and the male-to-female ratio is 2:1. LCH in children typically occurs around the age of 5 years. The disease affects the skull, orbit, mandible, palate, and long bones, as well as the lung, lymph nodes, spleen, and the central nervous system [26, 29]. Although craniofacial involvement is the most common manifestation both in adults and in children, orbital lesions are more frequently found in children while mandibular lesions are more frequently seen in adults. Reactivation episodes and death are more common in adults than in children while the 10-year overall survival is similar in both groups [30]. Treatment modalities include surgery, radiation therapy (RT), chemotherapy (ChT), and intralesional corticosteroid injection [31]. Low-dose irradiation has high rates of local control and symptomatic improvement. However, facial asymmetry is a feared long-term complication as RT impairs facial growth [32]. Clinical presentation in isolated maxillofacial lesions is often non-specific and includes pain, swelling, gingival hypertrophy, or tooth loosening. Conventional radiographs and CT reveal well-defined, punched-out radiolucent lesions with or without reactive sclerosis. A soft tissue mass surrounding the bony structures is equally present. A periosteal reaction (solid or sunburst type) has been reported in mandibular lesions [33], whereas a “hole-within-a-hole” sign and a button sequestrum are often observed in skull involvement (Fig. 3). The hole-within-a-hole sign corresponds to destruction of the inner and outer tables to differing degrees, therefore resulting in two overlying lesions of unequal size. MRI findings comprise low signal on T1, intermediate to high signal on T2, and variable contrast enhancement. ADC values appear to be higher in LCH than in malignant tumors [33]. In conjunction with the age of presentation, the combination of well-defined lesions on CT, high signal on T2, strong contrast enhancement, and ADC values >1.2 × 10^−3^ mm^2^/s is strongly evocative of LCH, especially when patients present with multifocal involvement or diabetes insipidus (Fig. 3). Although imaging findings can be strongly evocative of LCH, histology is mandatory.

Due to its high sensitivity, MRI is indispensable for the primary staging of LCH, as it enables to screen the entire body for bone involvement, to correctly assess the brain and to guide biopsy. As contrast enhancement on CEMRI has been shown to correlate with disease activity or perilesional inflammation, MRI is equally used to evaluate treatment response [27]. On FDG PET CT, LCH lesions are FDG avid (Fig. 3). However, due to the small size of some lesions, the overall sensitivity of FDG PET is limited (67%) [27]. The relatively high specificity of FDG PET for LCH (76% as opposed to only 47% with MRI) provides important information regarding disease response. Therefore, unnecessary treatment can be avoided [27]. Because combined MRI and PET CT are increasingly used in pediatric LCH, it has been suggested that PET MRI may play an important role for the initial evaluation of pediatric LCH and for facilitated monitoring of treatment response [16, 27, 34].

### Desmoid-type fibromatosis and desmoplastic fibroma

Desmoid-type fibromatosis also called desmoid tumor or aggressive fibromatosis is a rare histologically benign but locally aggressive tumor arising from the fascial and musculoaponeurotic tissues, lacking metastatic potential [35, 36]. It has an incidence of 0.2–0.4 per 100,000 cases per year [35] and may occasionally affect children. Desmoplastic fibroma (DF) is the intraosseous morphological mimic of desmoid-type fibromatosis [37]. The etiology of desmoid tumor and DF is unclear, and a genetic predisposition (Gardener syndrome and familial adenomatous polyposis) has been suggested. As in adults, pediatric desmoids and DFs may occur either sporadically or in association with familial adenomatous polyposis.

**Fig. 4:**
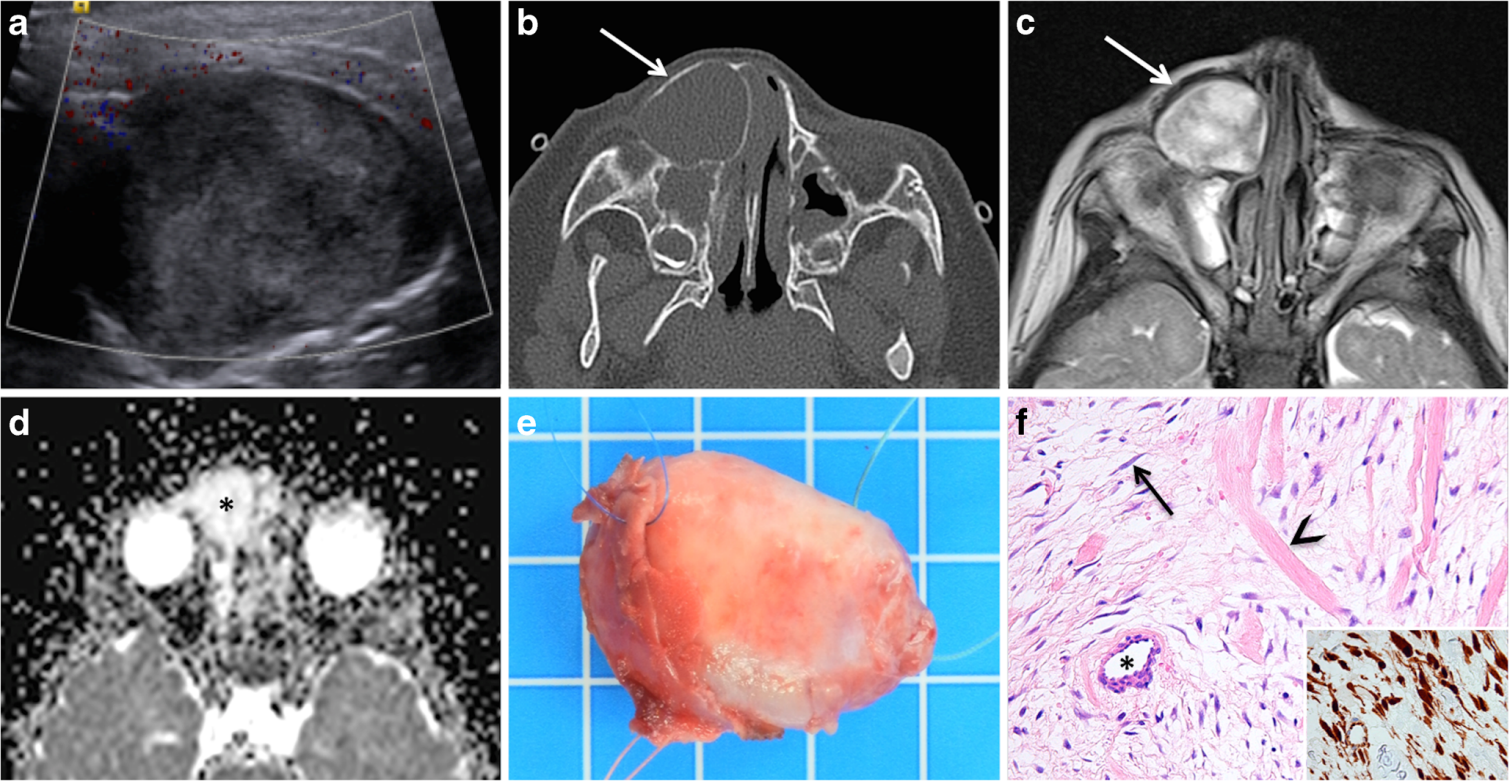
Desmoplastic fibroma (DF) in a 14-month-old girl with a rapidly growing facial mass. **a** Ultrasonography was non-specific revealing a solid heterogenous hypoechoic mass with apparently no vascularization suggesting the diagnosis of a dermoid/epidermoid cyst. **b** Axial CT (bone window) shows a well-delineated lesion with smooth bony expansion suggesting a mucocele or a benign tumor (*arrow*). **c**, **d** MRI findings typical of a benign tumor: predominantly high signal on axial T2 (*arrow* in **c**) and increased diffusion on the ADC map (*asterisk* in **d**). ADCmean = 1.9 × 10^−3^ mm^2^/s. **e** Photograph of gross surgical specimen: the lesion consisted of a 3.2-cm encapsulated firm nodule. **f** High-power photomicrograph (original magnification, ×200; H-E stain) shows a paucicellular proliferation of spindle- and stellate-shaped cells (*arrow*), embedded in a collagen-rich stroma (*arrowhead* indicates a thick collagen bundle); a thick-walled small artery with an open lumen is seen (*asterisk*). Immunohistochemistry (*inset* in **f**) shows cytoplasmic accumulation with nuclear delocalization of beta-catenin (original magnification, ×400, *brown color*). Despite morphological similarities with desmoid-type fibromatosis, Sanger sequencing revealed no mutation in the exon 3 of the *CTNNB1* gene encoding beta-catenin consistent with the diagnosis of desmoplastic fibroma

Beta-catenin is a cytoplasmic protein regulated by the adenomatous polyposis coli (APC) gene and is normally located beneath the cell membrane. Mutations in the CTNNB1 gene encoding beta-catenin and the APC gene cause beta-catenin delocalization and accumulation in the nuclei, demonstrated by immunohistochemistry. DFs only occasionally show beta catenin and, contrary to desmoid-type fibromatosis of soft tissue, do not display mutations in exon 3 of CTNNB1 [36]. Desmoid and DF have a high rate of post-surgical local recurrence, however no metastatic potential. According to some authors and based on observations in our institution, DF typically affects children between 18 months and 3 years of age and presents as a painless mass. Most DFs arise in the pelvis and long bones whereas DF arising within the mandible, within the maxilla, and in the cranium is rare [37, 38]. DF can occur either as a solitary lesion or as a manifestation of tuberous sclerosis. In fact, DF is considered to be one of the intraoral manifestations of tuberous sclerosis rather than a coincidental finding. Imaging findings in DF show an expansile intraosseous osteolytic lesion with non-aggressive looking borders, bone remodeling or scalloping, and imaging features suggesting a benign histology (Fig. 4). On MRI, the signal intensity on T2-weighted and STIR images and ADC values tend to be higher than in malignant tumors suggesting paucicellularity and benign histology (Fig. 4). Enhancement after intravenous gadolinium is often significant but does not provide additional information with respect to differential diagnosis; it is, however, important for tumor margin delineation in view of surgical resection. Lesions located in the calvarium or in skull base vicinity may be strongly adherent to the dura mater, and dural enhancement or dural thickening can be seen at imaging necessitating coagulation or removal of the dura during surgery. Surgical extirpation is the treatment of choice, and RT is indicated in incompletely excised or recurrent tumors. Close follow-up with cross-sectional imaging is essential as DF may recur.

### Inflammatory myofibroblastic tumor

**Fig. 5:**
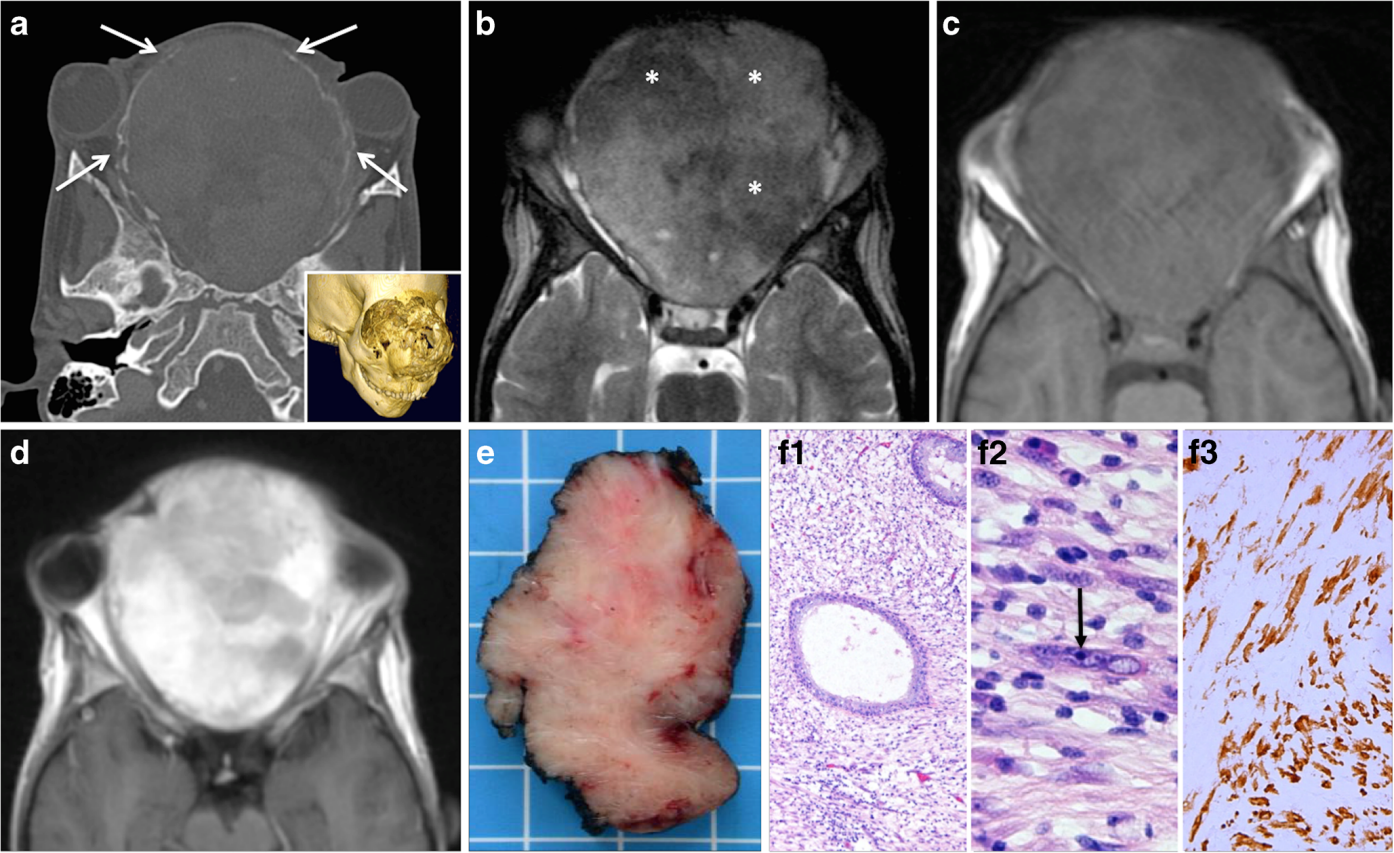
Inflammatory myofibroblastic tumor (IMT) in a 6-year-old girl with a rapidly growing mass and facial deformity. A rhabdomyosarcoma was suspected clinically. **a** Axial CT scan (bone window) shows an spherical midfacial lesion remodeling the facial skeleton, with a thin peripheral calcified rim (*arrows*), hypertelorism, and exophthalmos. Three-dimensional CT reconstruction (*inset* in **a**) shows “soap bubble appearance” with remodeling of the nasal cavity. **b** Axial T2 depicts multiple lobular predominantly hypointense tumor compartments (*asterisk*). Note that the T2 signal is quite homogeneous within each individual tumor compartment. **c** Axial T1 shows no areas of hemorrhage. **d** T1 post-contrast reveals strong homogenous enhancement within the different compartments. **e** Gross pathology: nodular fleshy to firm nodule. *f1* High-power photomicrograph (original magnification, ×200; H-E stain) showing proliferation of spindle cells arranged in a vaguely fascicular pattern, surrounding glands. At higher magnification (*f2*), mildly atypical myofibroblasts with prominent nucleoli (*arrow*); the accompanying inflammatory infiltrate is mainly composed of lymphocytes and plasma cells. *f3* On immunohistochemistry, the myofibroblastic spindle cells show strong cytoplasmic immunoreactivity for ALK protein (*brown color*), typical for IMT

Inflammatory myofibroblastic tumor (IMT) also previously referred to as inflammatory pseudotumor, as myofibroblastoma, or as plasma cell granuloma is an immunohistochemically diverse entity composed of myofibroblasts, small lymphocytes, and plasma cells. This rare and rather controversial tumor has both inflammatory and neoplastic characteristics including chromosomal alterations (translocations of the short arm of chromosome 2). The rearrangement of the anaplastic lymphoma kinase (ALK) gene at chromosome 2p23, which can be demonstrated by fluorescence in situ hybridization, is now regarded as pathognomonic for the definitive diagnosis. More than 50% of IMTs occur in children and adolescents, and there is a strong predilection for the lung, abdomen, retroperitoneum, and extremities [39]. In the HN, IMTs affect the orbit, larynx, oral cavity and oropharynx, parapharyngeal space, and thyroid gland. IMTs may also affect the paranasal sinuses, and on rare occasions, progression to overtly sarcomatous morphology or distant metastases can occur [40]. Because IMTs also tend to recur and because of the rare potential for malignant transformation, IMTs are classified as tumors with intermediate biological potential. IMTs often present as rapidly growing painless masses, and there are no systemic signs. Lesions arising in the paranasal sinuses and pterygopalatine fossa have a more aggressive behavior (Fig. 5) [41, 42].

Cross-sectional imaging is indispensable for treatment planning and for the assessment of precise tumor spread. In addition, it has been suggested that CT and MRI may help to differentiate between aggressive sinonasal malignancy and IMT. IMTs are most often lobular or spherical masses with a smooth, regular rim. The tumors are hyperdense on non-contrast-enhanced CT (NECT) and hypointense on T2, and they display strong enhancement after intravenous contrast material (Fig. 5). It has been suggested that the combination of hypointense signal on T2-weighted images and high ADC values is typical MRI features of IMT allowing a correct presumptive diagnosis [43]. Bone remodeling and thin peripheral calcification are further characteristics. However, when tumors grow very large, hemorrhage and necrosis may lead to heterogenous signal intensity on T2 rendering distinction from malignant lesions more difficult. The management of these tumors is challenging, and there are no generalized treatment protocols, especially for large lesions located in close proximity to vital structures. To date, complete surgical excision, corticosteroid treatment, or ChT with an ALK inhibitor in refractory cases is recommended, corticosteroid treatment being the most common treatment modality in smaller lesions.

### Juvenile ossifying fibroma

**Fig. 6:**
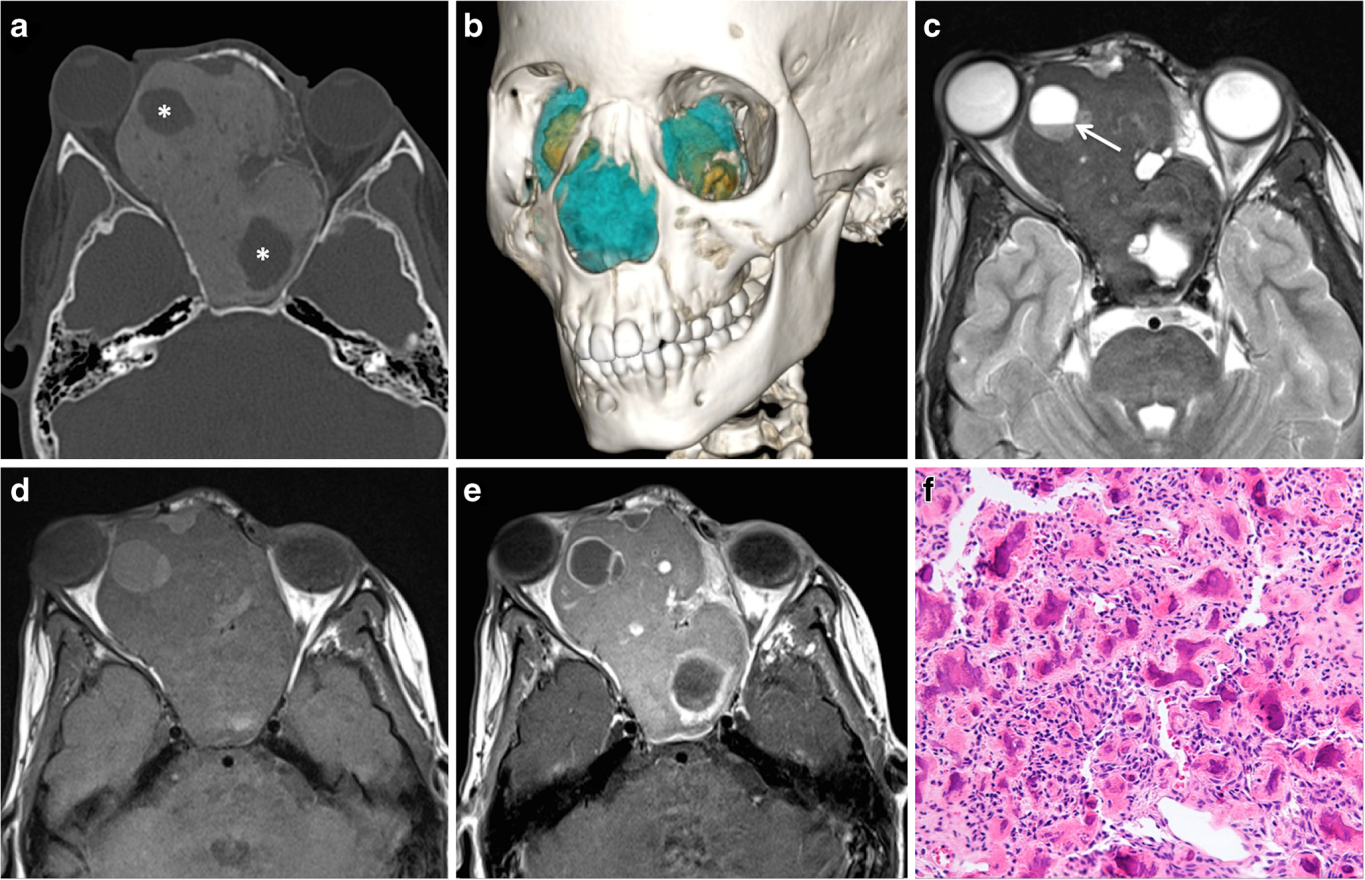
Juvenile psammomatoid ossifying fibroma (JPOF) in a 10-year-old girl with periorbital swelling and hypertelorism. **a** Axial CT scan (bone window) shows an expansile, well-circumscribed spherical lesion with ground glass opacity and rounded cystic areas (*asterisks*). Invasion of ethmoid and sphenoid sinuses and orbits. Lateral displacement of the right globe with proptosis. **b** 3D CT reconstruction: lesion in *blue* and cystic areas in *yellow*. **c** Axial T2 reveals that the solid parts with ground glass opacity on CT have a very low T2 signal. The cystic areas have fluid-fluid levels (*arrow*). **d** Axial T1 shows homogenous low signal of the mass matrix with increased signal in the cystic components indicating proteinaceous or hemorrhagic content. **e** Axial post-contrast T1 shows moderate homogenous matrix enhancement and rim enhancement of the cystic areas. **f** High-power photomicrograph (original magnification, ×200; H-E stain) shows small concentric lamellated ossicles resembling psammoma bodies surrounded by a thick irregular collagenous rim, embedded in a fibroblastic stroma

Juvenile ossifying fibroma is a rare benign fibro-osseous neoplasm usually seen in the craniofacial bones. It has the potential for aggressive growth as the denomination previously used by the WHO (juvenile aggressive ossifying fibroma) implies. Two distinct histological variants have been described: juvenile psammomatoid ossifying fibroma (JPOF) and juvenile trabecular ossifying fibroma (JTOF) [44, 45]. Both neoplasms have different age distributions, different predilection sites, and different radiologic features. While JPOF is more common in adults in the paranasal sinuses (ethmoid and sphenoid), JTOF is predominantly seen in children between 8 and 10 years of age with a predilection for the alveolar ridge of the maxilla. JPOF often presents with major facial deformation and occasionally hypertelorism due to bony expansion (Fig. 6). Histologically, JPOF shows numerous psammomatous calcifications, which account for the ground glass opacity on CT. The radiologic aspect is characteristic and allows the correct diagnosis in most cases (Fig. 6). JPOF presents as an expansile mass with well-defined sclerotic borders, with ground glass opacity, and with areas of central radiolucency. These radiolucent areas have fluid-fluid levels easily seen on MRI. The tumor shows displacement rather than infiltration of soft tissues, and the associated bony expansion has a typical spherical configuration (Fig. 6). The differential diagnosis is fibrous dysplasia (FD). However, in contrast to JPOF, FD typically shows ill-defined borders blending with the normal bone and the overall shape of the affected bone is preserved. In most patients with FD, CT reveals characteristic bony expansion with a “ground glass” appearance or mixed radiolucency, widened diploic space with outer table displacement or bubbling skull vault lesions. On PET CT, both IPOF and FD may show high FDG uptake, thereby mimicking bone metastases or other aggressive bone lesions [46]. However, the characteristic CT aspect and—if necessary—the high ADC values help to avoid misinterpretation. Therefore, correlation between FDG uptake on PET CT and morphologic CT or MRI features is essential. As treatment consists of surgery, cross-sectional imaging plays an important role not only for diagnosis but also for precise planning of resection. Multimodality image fusion and 3D volume rendering are helpful for surgical resection.

### Ewing sarcoma

**Fig. 7:**
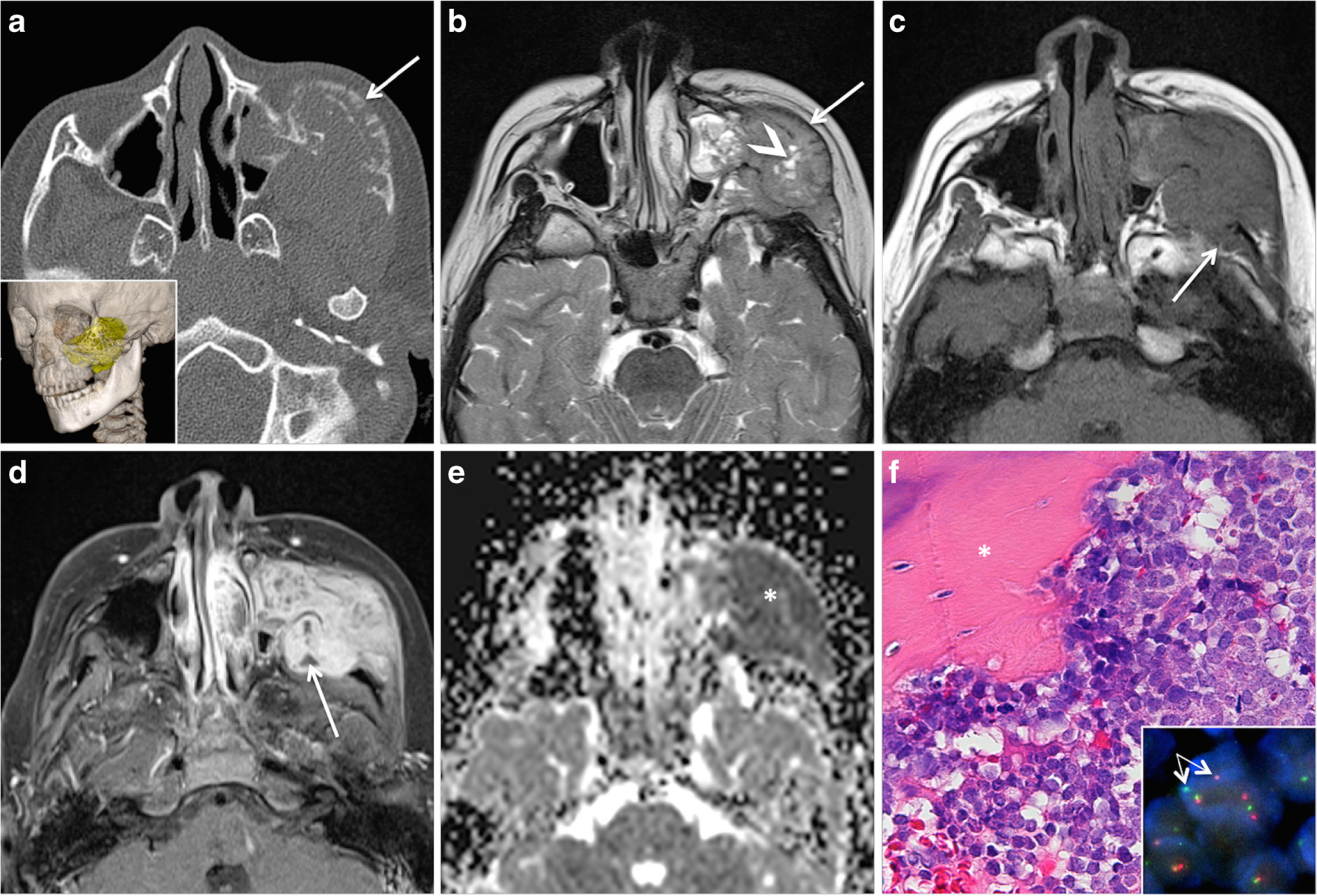
Ewing sarcoma (ES) of the maxilla: characteristic findings in a 5-year-old girl with gradually progressing indurated swelling of the left cheek. **a** Axial CT scan (bone window) shows a lytic lesion with sunburst-type periosteal reaction and bone expansion (*arrow*). Three-dimensional CT reconstruction (*inset* in **a**) illustrates invasion of the left maxillary sinus, orbital floor, zygomatic arch, and maxillary alveolar ridge. The tumor is rendered in *yellow*. **b** Axial T2 shows intermediate tumor signal with small areas of necrosis (bright areas, *arrowhead*), invasion of the left maxillary sinus, and zygomatic arch (*arrow*), as well as invasion of the suprazygomatic masticator space. **c** Axial T1 depicts non-specific intermediate signal of the mass with invasion of the temporalis muscle (*arrow*). **d** Axial post-contrast fat-saturated T1 reveals inhomogeneous and strong enhancement with small necrotic areas (*arrow*). **e** Restricted diffusion (*asterisk*) with very low signal on the ADC map (ADC = 0.6 × 10^−3^ mm^2^/s). **f** High-power photomicrograph (original magnification, ×400; H-E stain) shows that the tumor is composed of sheets of undifferentiated small round blue cells, invading bone (*asterisk*). Immunohistochemistry (not shown) reveals membranous reactivity to CD99 (O13 mouse monoclonal antibody). Fluorescence in situ hybridization (FISH) shows EWSR1 gene rearrangement (*inset* in **f**, *arrows*; Vysis®, LSI dual-color break-apart probe FISH probe); translocation results in the separation of a pair of *green* and *orange* signals. Reverse transcription polymerase chain reaction (RT-PCR) uncovered a t(11;22)(q24;q21)*EWSR1-FLI1* translocation, thereby further confirming ES

Ewing sarcoma (ES) is a rare primary bone malignancy mainly affecting children and adolescents, most cases occurring between 10 and 20 years of age [47–49]. It arises in the medullary cavity of bone and invades the Haversian system. The tumor is composed of narrow sheets of small, round, and densely packed cells with scanty cytoplasm, showing varying degrees of neuroectodermal differentiation. These small blue rounded cells are glycogen positive, and on electron microscopy, features of neural differentiation are found. The origin of ES is a subject of much debate, and it is not yet clear whether ES arises from primitive neuroectodermal cells or from mesenchymal stem cells [49]. In almost all ES cases, there is a characteristic reciprocal chromosomal translocation between the EWSR1 gene and a gene of the ES tumor family of transcription factors. The ES tumor family comprises ES, neuroepithelioma, peripheral primitive neuroectodermal tumor, and Askin tumor. As a highly malignant tumor, ES has an early metastatic potential to the lungs, liver, bone marrow, and other organs [48]. Only 2% of all ES cases arise in the skull and face, and involvement of the zygoma is very rare [47]. Clinical presentation in the HN is non-specific and includes pain, facial swelling, palpable mass, fever, and increased erythrocyte sedimentation rate (ESR). However, on cross-sectional imaging, ES presents as a large poorly defined lesion with extension into the surrounding soft tissues. Permeative osteolysis, irregular areas of sclerosis and an onion skin periosteal reaction are common on CT. Sunburst appearance, Codman triangle, bony expansion, and the presence of a soft tissue mass are further features suggesting an aggressive tumor (Fig. 7). On T2-weighted and contrast-enhanced T1-weighted images, ES displays heterogeneous signal, and on DWI, there is marked restriction of diffusion with low ADC values (<0.9–1 × 10^−3^ mm^2^/s) in keeping with the malignant nature of the tumor (Fig. 7). In some ES cases of the jaws, distinction from chronic osteomyelitis (OM) can be challenging on the basis of clinical findings (demographics, history, physical examination, and laboratory findings) and on the basis of cross-sectional imaging features. As recently suggested, other than ethnicity (African Americans are more likely to have OM than ES), no clinical feature improves diagnostic accuracy while among the morphologic features (without DWI), only the presence of a soft tissue mass at MRI appears to be significantly associated with ES [50]. On FDG PET CT, both OM and ES show increased tracer uptake within the bone and within the surrounding soft tissues due to increased glucose metabolism in inflammatory and malignant tissue, respectively.

The metastatic workup in ES includes whole-body MRI with DWI and FDG PET CT. Treatment consists of ChT with additional surgery depending on the location of the tumor. Survival depends upon the presence of distant metastases, primary tumor location, and red blood cell count [51]. The use of DWI is important not only for diagnostic and staging purposes but also for the monitoring of treatment response and for personalized therapeutic approaches. For example, involved compartment irradiation detected by whole-body MRI combined with high-dose ChT and stem cell rescue in ES patients with multiple primary bone metastases appears to significantly improve survival at 5 and 10 years after diagnosis in comparison to ES patients treated according to the standard protocol of the European Intergroup Cooperative Ewing Sarcoma Study-92 [52]. On follow-up after neoadjuvant ChT, good responders typically show a significant and early increase of ADC values corresponding to treatment-related tumor necrosis while morphologic features including contrast-to-noise ratio on T2 and on gadolinium-enhanced T1 tend to remain similar. A recent study found that 3D tumor measurements are better predictors of the therapeutic response in ES than in one-dimensional (1D) Response Evaluation Criteria in Solid Tumors (RECIST) and 2D measurements (WHO criteria); 3D measurements also showed a significantly higher correlation with clinical outcomes [53].

Although the mean radiation dose of patients examined with FDG PET CT for staging and treatment of ES is on average 23% higher than for patients not examined with PET CT [54], whole-body PET CT has a relevant impact on therapy planning in children outperforming conventional imaging modalities (US, CT, bone scintigraphy, and morphologic MRI). FDG PET and conventional imaging modalities appear to be equally effective in the detection of primary tumors; however, PET is superior for the detection of lymph node metastases and bone metastases, whereas CT is more reliable for the depiction of lung metastases [55]. Quantification of FDG uptake with SUV may also be able to predict prognosis of children with ES: patients with a pretreatment SUVmax ≤5.8 have been shown to survive significantly longer than those with a SUVmax >5.8 [56].

### Primary intraosseous lymphoma

**Fig. 8:**
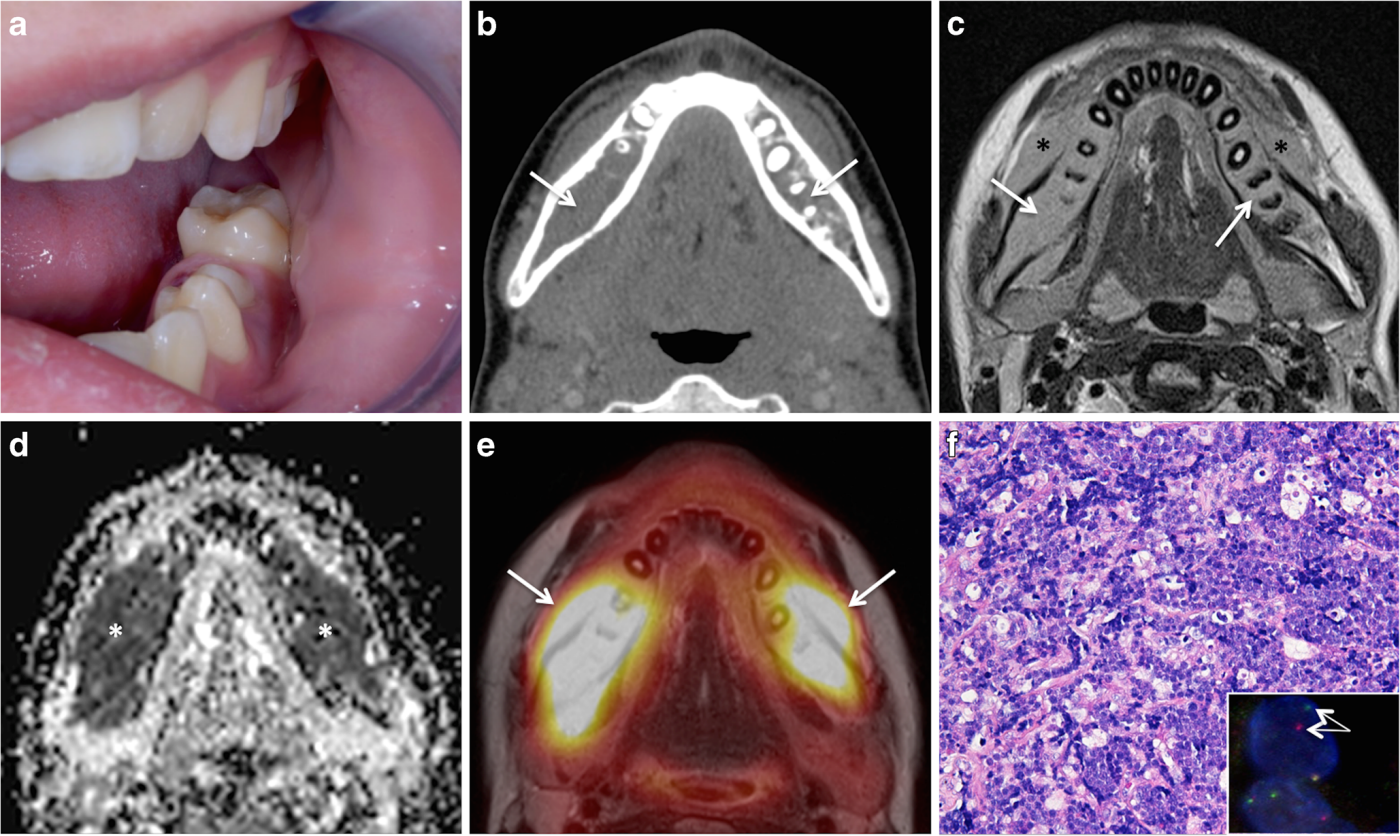
Primary intraosseous Burkitt lymphoma in a 14-year-old boy with gingival and vestibular swelling and increased molar teeth mobility. **a** Clinical aspect: submucosal swelling with intact mucosa. Courtesy: Andrej Terzic, MD, Clinic for maxillofacial surgery, University Hospital Geneva. **b** Axial contrast-enhanced CT shows diffuse marrow infiltration bilaterally (*arrows*) and absent bone trabeculae. **c** Axial T2 reveals homogeneous signal (*arrows*) and root resorption on the *right*. Note extraosseous bilateral spread (*asterisk*). **d** Very low ADC values (0.3–0.5 × 10^−3^ mm^2^/s) (*asterisk*) typical of lymphoma. **e** Axial PET MRI (PET fused with T2) shows high intraosseous and extraosseous FDG uptake with very high SUVs (SUVmean/SUVmax = 14.32/19.98) (*arrows*). **f** High-power photomicrograph (original magnification, ×200; H-E stain) shows sheets of undifferentiated medium-sized lymphoid cells and intermingled tangible body macrophages, creating a characteristic “starry sky” appearance. Upon immunohistochemical evaluation, the tumor cells showed reactivity to CD20, CD10, CD79A, BCL6, and MYC, and the proliferation index was close to 100% (not shown). A fluorescent in situ hybridization (FISH) assay (*inset* in **f**) confirmed *MYC* gene rearrangement. Translocation results in the separation of a pair of *green* and *orange* signals (*arrows*) (Vysis®, LSI dual-color break-apart probe)

Non-Hodgkin’s lymphoma (NHL) tends to involve extranodal sites more often than Hodgkin’s lymphoma [57, 58]. The most common pediatric NHL types in the HN include diffuse large B cell-type NHL, lymphoblastic lymphoma, and Burkitt lymphoma. Epstein-Barr virus (EBV) infection in early life is associated not only with Burkitt lymphoma but also with Hodgkin’s lymphoma, nasopharyngeal carcinoma, and post-transplantation lymphoproliferative disease [58]. Endemic Burkitt lymphoma, which is mainly seen in equatorial Africa, has a 95% rate of association with EBV and is often linked to coinfection with *Plasmodium falciparum* [58]. Sporadic Burkitt lymphoma is associated with EBV infection in up to 30% of cases, and HIV-associated Burkitt lymphoma is positive for EBV in up to 50%. Whenever lymphoma arises within a bone without nodal or visceral involvement within 6 months of presentation, it is named primary intraosseous lymphoma (PIL) [57]. According to the literature, PIL constitutes about 3% of all malignant bone tumors and 5% of all extranodal lymphomas. Most PILs are Burkitt lymphomas. PIL is rare in children under 10 years of age. Pediatric PIL most often involves the lower extremities whereas mandibular or maxillary involvement is rare. Unfortunately, most PILs affecting the jaws are often misdiagnosed, as patients may present with tooth pain or tooth mobility, gingival swelling, and bleeding, which can mimic periodontal disease. The initial wrong diagnosis is often followed by multiple root canal treatments or extraction of wisdom teeth. However, the diagnosis of maxillofacial PIL can be strongly suggested by radiologists on the basis of MRI findings, thereby contributing to a rapid diagnosis (Fig. 8). On NECT, PIL demonstrates diffuse permeative osteolysis, cortical destruction, and juxtacortical soft tissue masses. The marrow space of the affected bone has low signal intensity on T1 and intermediate signal intensity on T2. On contrast-enhanced T1, PIL displays homogenous and diffuse enhancement. In association with very low ADC values (ADC = 0.4–0.7 × 10^−3^ mm^2^/s), the diagnosis can be suggested with a high degree of confidence, therefore prompting rapid biopsy. Whole-body staging is necessary for the diagnosis of PIL, as involvement of other sites needs to be ruled out and PIL needs to be distinguished from bone deposits caused by disseminated NHL. ChT is the treatment of choice, and prognosis is usually good. In a recent study dealing with primary pediatric lymphoma of the bone, post-treatment follow-up with conventional morphologic imaging (without DWI) did not alter clinical management in the absence of symptoms, in particular also due to the lack of correlation between osseous changes after treatment on conventional morphologic images and clinical outcome [59]. Only FDG PET CT proved capable of demonstrating metabolic imaging changes consistent with the clinical response to treatment [59]. The diagnosis of PIL is, therefore, most often complemented by FDG PET CT or more recently by FDG PET MRI.

## Metastases to the facial bones

Metastases to the facial bones are very rare in children. They typically occur in widespread disease, and the primary tumor is most often known. However, whenever bone metastases without a known primary tumor are considered in the differential diagnosis on the basis of cross-sectional imaging, possible primaries would include neuroblastoma, ES, angiosarcoma, retinoblastoma, NHL, and leukemia.

### Neuroblastoma

**Fig. 9:**
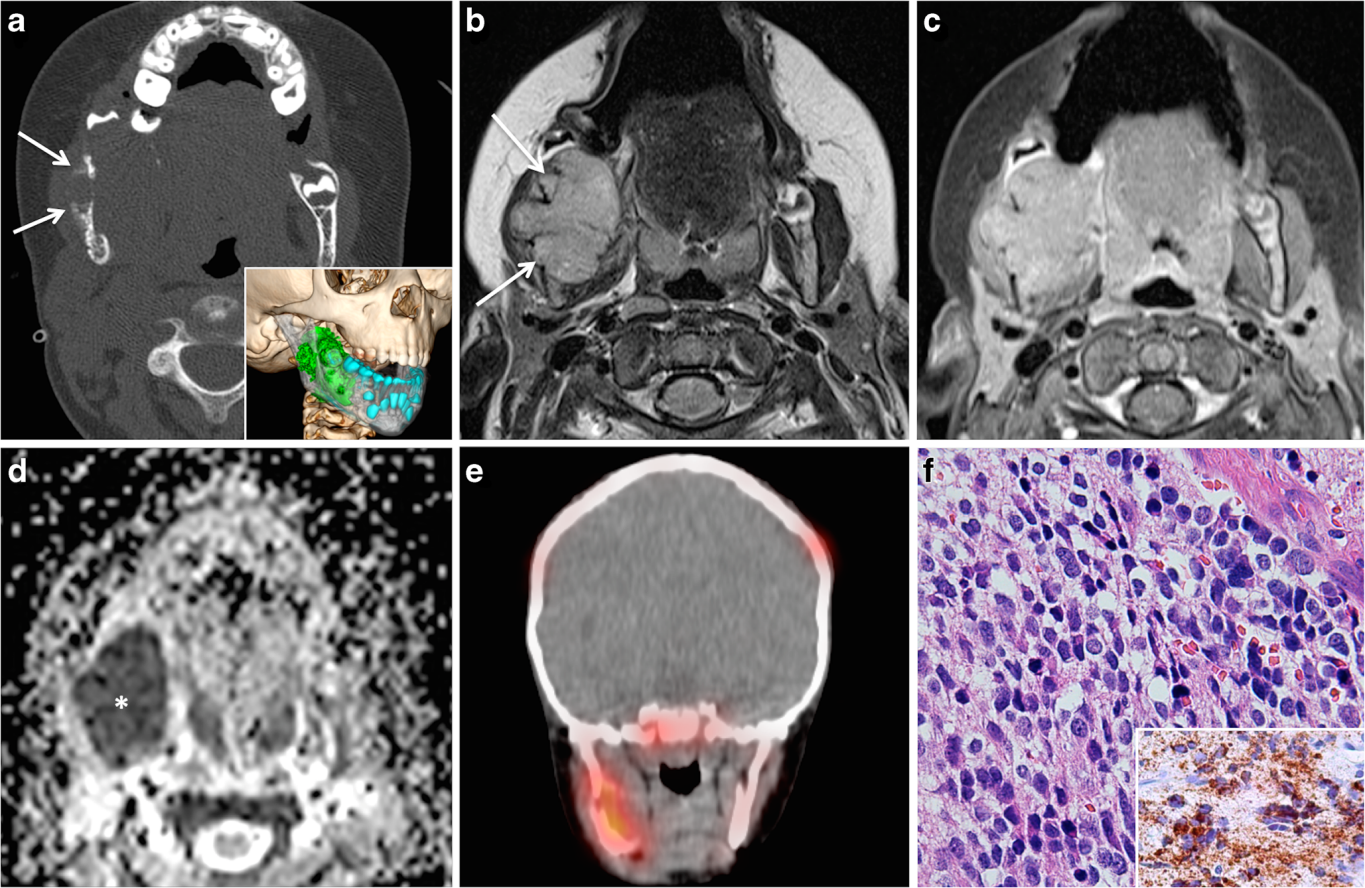
Mandibular metastasis from neuroblastoma (NB) in a 5-year-old girl with fatigue and localized right mandibular swelling. Treated for abdominal NB. **a** Axial CT scan (bone window) shows extensive osteolysis of the mandibular angle with ill-defined, destructive margins, overlying soft tissue mass, expanded cortex with Codman triangle typical of an aggressive bone lesion (*arrows*). Three-dimensional CT reconstruction (*inset* in **a**) illustrates the relationship between the tumor (*green*), inferior alveolar nerve canal (*gray*), and teeth (*blue*). **b** Axial T2 reveals polypoid lesion with intermediate signal (*arrows*) and major extraosseous involvement. Invasion of the masseter muscle. **c** Homogeneous non-specific enhancement on axial post-contrast fat-saturated T1. **d** Restricted diffusion with very low signal on the ADC map (*asterisk*) (ADC = 0.54 × 10^−3^ mm^2^/s). Imaging findings are characteristic of a highly malignant tumor and—in this clinical context—suggest metastasis from NB. Whole-body ^123^I–MIBG SPECT/CT (**e**) revealed further calvarial and skull base metastases. **f** High-power photomicrograph (original magnification, ×400; H-E stain) shows poorly differentiated neuroblasts surrounded by small amounts of neuropil (finely fibrillar and eosinophilic matrix corresponding to neuritic processes) and characteristic reactivity to NB84 on immunohistochemistry (*inset* in **f**)

Neuroblastoma (NB) is the most common solid childhood malignancy and accounts for about 8% of all childhood cancers in industrialized countries. It typically occurs in infants and children under the age of 5, most cases being seen under the age of 2 years. The tumor arises from pluripotent sympathetic cells located in the sympathetic ganglia, adrenal medulla, and other sites [60]. In infants, most NBs arise in the chest, whereas in children, most NBs arise in the abdomen. NBs have many chromosomal and molecular abnormalities, such as amplification of the MYCN oncogene (more common in advanced stage disease); overexpression of the HRAS oncogene (more common in lower-stage disease); chromosome 1p deletion; allelic losses of chromosomes 11q, 14q, and 17q; and variable expression of neurotrophin receptor gene products. Prognosis in NB depends upon the age of presentation, stage, and molecular factors. Patients with low to intermediate risk NB have an excellent prognosis while those with high-risk tumors have a very poor survival despite intensive ChT, RT, and autologous bone marrow transplantation [61]. Most NB lesions seen in the HN are metastases from abdominal NB. Unfortunately, metastatic disease is very common among children older than 18 months of age. According to a recent study, NB was the most common tumor with metastases to the jaws in the age group ranging from 4 months to 16 years and metastasis to the maxilla or mandible was found to be the first metastatic site in half of these patients [62, 63]. On CT and MRI, NB metastases to the maxillofacial skeleton have all imaging characteristics of aggressive tumors (Fig. 9): diffuse permeative or moth-eaten osteolysis, cortical destruction, poorly delineated soft tissue masses with or without calcification, and areas of necrosis. On MRI, NB shows intermediate signal intensity on T2, strong enhancement after intravenous administration of gadolinium, and low ADC values in the solid portions. Areas of necrosis are common.

In addition to CT and MRI, metaiodobenzylguanidine labeled to iodine^123^ (^123^I MIBG) scintigraphy, FDG, and fluoro-dihydroxyphenylalanine (FDOPA) PET CT are used for the assessment of primary and metastatic NB. ^123^I MIBG is a specific guanethidine derivative that is taken up by the norepinephrine transporter (NET) on NB cells. The reported sensitivity of ^123^I MIBG scintigraphy for the detection of NB and metastases varies from 67 to 100% with a 10% rate of false-negative evaluations, as the tumor may not show tracer accumulation [64]. For these patients, FDG PET CT has been recommended for staging purposes and for assessing response to therapy. However, data on the performance of PET CT in comparison to other imaging modalities are currently quite limited. Nevertheless, FDG and FDOPA PET CT can be used to stratify patients with NB into risk groups [65]. FDG SUVmax ≥3.31 and FDOPA SUVmax <4.12 have been identified as ultra-high-risk features capable of distinguishing the most unfavorable genomic types (MYCN amplification and segmental chromosomal alterations) with a sensitivity of 81% and a specificity of 93% [65]. Therefore, the ratio between FDG and FDOPA uptake may complement the current risk stratification systems of NB [65]. Treatment of symptomatic metastases to the facial bones, in particular the mandible, is often done with short intensive RT courses to palliate pain [66]. Short RT courses are more suitable for the young patients especially if general anesthesia is needed.

## Conclusion

Interpretation of imaging findings in children with non-odontogenic tumors of the facial skeleton is challenging. Multimodality imaging allows for detailed evaluation of anatomic extent and increases diagnostic confidence. It also contributes important information for prognosis and risk stratification and enhances pretherapeutic assessment, as well as monitoring of patients after treatment. This review article summarizes multimodality imaging features of these rare tumors.

## Abbreviations

ADC: Apparent diffusion coefficient
CEMRI: Contrast-enhanced MRI
ChT: Chemotherapy
CBCT: Cone beam computed tomography
CT: Computed tomography
DF: Desmoplastic fibroma
DWI: Diffusion-weighted imaging
EBV: Epstein-Barr virus
EG: Eosinophilic granuloma
ESR: Erythrocyte sedimentation rate
ES: Ewing sarcoma
FD: Fibrous dysplasia
FDG: F18-Fluoro-deoxy-d-glucose
FDOPA: F18-Fluoro-dihydroxyphenylalanine
H-E: Hematoxylin and eosin staining
HN: Head and neck
IMT: Inflammatory myofibroblastic tumor
JPOF: Juvenile psammomatoid ossifying fibroma
JTOF: Juvenile trabecular ossifying fibroma
LCH: Langerhans cell histiocytosis
MRI: Magnetic resonance imaging
NB: Neuroblastoma
NHL: Non-Hodgkin’s lymphoma
NECT: Non-contrast-enhanced CT
OM: Osteomyelitis
PET CT: Positron emission tomography computed tomography
RECIST: Response Evaluation Criteria in Solid Tumors
RT: Radiotherapy
US: Ultrasonography
WHO: World Health Organization
